# Effects of a Short-Term Left Ventricular Assist Device on Hemodynamics in a Heart Failure Patient-Specific Aorta Model: A CFD Study

**DOI:** 10.3389/fphys.2021.733464

**Published:** 2021-09-21

**Authors:** Yu Wang, Junwei Wang, Jing Peng, Mingming Huo, Zhiqiang Yang, Guruprasad A. Giridharan, Yong Luan, Kairong Qin

**Affiliations:** ^1^School of Optoelectronic Engineering and Instrumentation Science, Dalian University of Technology, Dalian, China; ^2^Department of Cardiovascular Computed Tomography (CT) Examination, The First Affiliated Hospital of Dalian Medical University, Dalian, China; ^3^Department of Bioengineering, University of Louisville, Louisville, KY, United States; ^4^Department of Anesthesiology, The First Affiliated Hospital of Dalian Medical University, Dalian, China

**Keywords:** CFD, hemodynamic performance, patient-specific aorta model, left ventricular assist device, heart failure

## Abstract

Patients with heart failure (HF) or undergoing cardiogenic shock and percutaneous coronary intervention require short-term cardiac support. Short-term cardiac support using a left ventricular assist device (LVAD) alters the pressure and flows of the vasculature by enhancing perfusion and improving the hemodynamic performance for the HF patients. However, due to the position of the inflow and outflow of the LVAD, the local hemodynamics within the aorta is altered with the LVAD support. Specifically, blood velocity, wall shear stress, and pressure difference are altered within the aorta. In this study, computational fluid dynamics (CFD) was used to elucidate the effects of a short-term LVAD for hemodynamic performance in a patient-specific aorta model. The three-dimensional (3D) geometric models of a patient-specific aorta and a short-term LVAD, Impella CP, were created. Velocity, wall shear stress, and pressure difference in the patient-specific aorta model with the Impella CP assistance were calculated and compared with the baseline values of the aorta without Impella CP support. Impella CP support augmented cardiac output, blood velocity, wall shear stress, and pressure difference in the aorta. The proposed CFD study could analyze the quantitative changes in the important hemodynamic parameters while considering the effects of Impella CP, and provide a scientific basis for further predicting and assessing the effects of these hemodynamic signals on the aorta.

## Introduction

Cardiovascular diseases are the leading cause of death and they account for 56% of total mortality in the modern era. Among such diseases, the number of patients with heart failure (HF) has exceeded 20 million worldwide, and is increasing at an annual rate of about 2 million people (Benjamin et al., 2017). HF refers to the clinical syndrome of circulatory disorder caused by the obstruction of systolic and/or diastolic functions of the heart, which cannot fully discharge the amount of venous return to the heart (Doehner et al., 2018; Van der Meer et al., 2019). HF is not an independent disease, but a chronic and progressive disease and the end stage of the development of cardiovascular diseases (Koshy et al., 2020; McEwan et al., 2020). Therefore, ventricular geometry transition can be observed associated with the end-stage HF (Li et al., 2020; Guan et al., 2021; Shavik et al., 2021). Moreover, hemodynamic variables such as flow pattern, wall shear stress (WSS), and distribution of blood pressure difference (Δ*P*) in the aorta could also be aberrant with the development of HF, causing vascular and arterial remodeling (Nakamura, 1999; Ky et al., 2013; Leite et al., 2019), and eventually inducing vascular and aortic diseases such as aorta coarctation, aortic valve stenosis, aortic stiffness, aortic insufficiency, etc. (Reddy et al., 2017; Nagao et al., 2018; Keen and Johnson, 2019). Therefore, quantitatively studying hemodynamic behaviors in the aorta regarding the geometric structure and functional characteristics is of great significance for the diagnosis and treatment of HF.

Furthermore, since HF is generally not curable but treatable, left ventricular assist devices (LVADs), as life-saving medical devices, have been successfully developed for the HF treatment (Slaughter et al., 2009; Schumer et al., 2016; Rogers et al., 2017). Durable LVAD support has been initiated in patients with chronic HF as it is superior to optimal medical management. Although heart transplantation has the best outcomes, there is an extreme lack of availability of heart donors in the world (Simaan et al., 2009). In addition, patients who are under acute cardiogenic shock or patients with imminent HF due to coronary heart disease are provided with short-term LVAD as a bridge to a decision or hemodynamic support during the percutaneous coronary intervention (PCI; i.e., stents) to mitigate cardiac hypoxia. LVADs can enhance perfusion and generally improve hemodynamic status by lowering the left ventricular work and increasing aortic pressure and flow. However, the local hemodynamic effects of the LVADs on aorta, especially the fluid velocity profile, WSS, and Δ*P*, are altered due to the outlet of the LVAD. Thus, it would be necessary to numerically analyze hemodynamics of the aorta for patients implanted with the LVADs.

To analyze hemodynamics in the aorta quantitatively, analyzing the fluid-dynamic variables is essential. However, traditional strategies using lumped parameter models using *in vitro* even *in vivo* studies are not adequate to fully understand geometric and functional properties and fluid-dynamic of the aorta (Gramigna et al., 2015; Carnahan et al., 2017; Wang et al., 2017). A crucial challenge facing such studies is that usually high fidelity pressure and/or flow measurements for hemodynamic analysis are extremely difficult because of the complex geometry and small dimensions of the aortic arch and blood vessels (Mazzitelli et al., 2016). These measurements also require implantation of sensors, which require invasive thoracotomy or sternotomy and is not feasible in patients due to the higher risk of mortality. Therefore, the hemodynamics in the aorta was investigated *via* computational fluid dynamics (CFD).

Different CFD; models have been established to quantitatively analyze the hemodynamics in the aorta under varying physiological conditions. For instance, studies by coupling CFD and some medical imaging technologies like Doppler echocardiography and magnetic resonance imaging (MRI) effectively predicted aneurysm and coarctation of the aorta (Brüning et al., 2018; Perinajová et al., 2021). Compared with MRI or computed tomography (CT), some expected dynamic behaviors of aortic diseases under stressed conditions can be better assessed *via* CFD modeling (Osswald et al., 2017; Febina et al., 2018), which can also be used to analyze the formation mechanism of aortic diseases (Zhang X. et al., 2020). However, most HF-related studies did not model the effects of LVAD support on the aorta. There have been literature studies demonstrating the changes in hemodynamics due to LVAD support using lumped parameter models, but these models are incapable of elucidating the local effects of the LVAD support. CFD is necessary to obtain the local distribution of some key parameters such as WSS, velocity vectors, etc. A few CFD studies combining the aorta and LVADs were based on the long-term, durable, and surgically-implanted LVADs (Mazzitelli et al., 2016; Yoshida et al., 2020), which have different outlets and outflows compared with short-term percutaneously inserted devices. Furthermore, short-term LVADs are used for patients with different indications compared with durable LVADs. For instance, Impella, one class of short-term LVADs, is a microaxial and catheter-mounted blood pump, which is percutaneously inserted into the aorta. Impella is indicated for circulatory support to patients with cardiogenic shock and high-risk PCI (Schrage et al., 2019; Amin et al., 2020), who do not always have significant remodeling of the heart and aorta due to prolonged and chronic HF. Therefore, the hemodynamic effects of the aorta in conjunction with a short-term blood pump need to be further studied.

In this work, the CFD simulation is implemented using a patient-specific aorta model by adding the effects of a short-term blood pump (Impella CP), in order to better understand the local hemodynamic effects in the aorta. Velocity, WSS, and Δ*P* distribution in the aorta are analyzed and also compared with the results only with the patient-specific aorta model without Impella CP support.

## Materials and Methods

### Governing Equations

In this CFD simulation, blood is assumed to be the incompressible Newtonian fluid. The governing equations for blood flows are set as follows (Deissler, 1984):


(1) $$\frac{\partial {\overset{¯}{V}}_{i}}{\partial t}+{\overset{¯}{V}}_{j}\frac{\partial {\overset{¯}{V}}_{i}}{\partial x_{j}}=-\frac{1}{\rho }\frac{\partial \overset{¯}{p}}{\partial x_{i}}+\frac{\mu }{\rho }\frac{\partial^{2}{\overset{¯}{V}}_{i}}{\partial x_{i}\partial x_{j}}-\frac{\bar{\partial {V^{\prime }}_{i}{V^{\prime }}_{j}}}{\partial x_{j}}$$

(2) $$\begin{array}{l} \frac{\partial {\bar{V}}_{i}}{\partial x_{i}}=0 \end{array}$$

where ${\bar{V}}_{i}$ (*i* = 1, 2, 3) represents the average velocities, *t* is the time, *x*_*i*_ represents the space coordinates in three-dimensional Cartesian coordinate system, μ is the dynamic viscosity at 0.004 kg/m^−1^/s^−1^, ρ is the fluid density at 1,060 kg/m^3^, $\bar{p}$ is the average pressure. The second item on the right side in Equation (1) reflects the viscous effect, shear stress, and ${\bar{\tau }}_{ij}$, can be rewritten as:


(3) $$\begin{array}{l} {\bar{\tau }}_{ij}\;=\;\mu \frac{\partial {\bar{V}}_{i}}{\partial x_{j}} \end{array}$$

The last term in Equation (1) is called Reynolds stress term. Since this term is unknown, the shear stress transport *k*–ω turbulence model is used for the CFD simulation (Menter, 1994). The turbulent kinetic energy transport equation and specific dissipation rate transport equation are as follows (Qiao et al., 2016):


(4) $$\rho \frac{\partial k}{\partial t}+\rho \frac{\partial (k{\overset{¯}{V}}_{i})}{\partial x_{i}}=\frac{\partial }{\partial x_{j}}(\Gamma_{k}\frac{\partial k}{\partial x_{j}})+G_{k}-Y_{k}$$

(5) $$\rho \frac{\partial \omega }{\partial t}+\rho \frac{\partial (\omega {\overset{¯}{V}}_{i})}{\partial x_{i}}=\frac{\partial }{\partial x_{j}}(\Gamma_{\omega }\frac{\partial \omega }{\partial x_{j}})+G_{\omega }-Y_{\omega }$$

where *k* and ω represent the turbulent scalar, called turbulent kinetic energy and specific dissipation rate, respectively. *G*_*k*_ is the turbulent kinetic energy produced by laminar velocity gradient, *G*_ω_ is the turbulent kinetic energy generated by equation (5). Γ_*k*_ and Γ_ω_ represent the effective diffusion terms of *k* and ω, respectively; *Y*_*k*_ and *Y*_ω_ represent turbulence due to the diffusion.

### Geometric Model of the Patient-Specific Aorta

**Figure 1:**
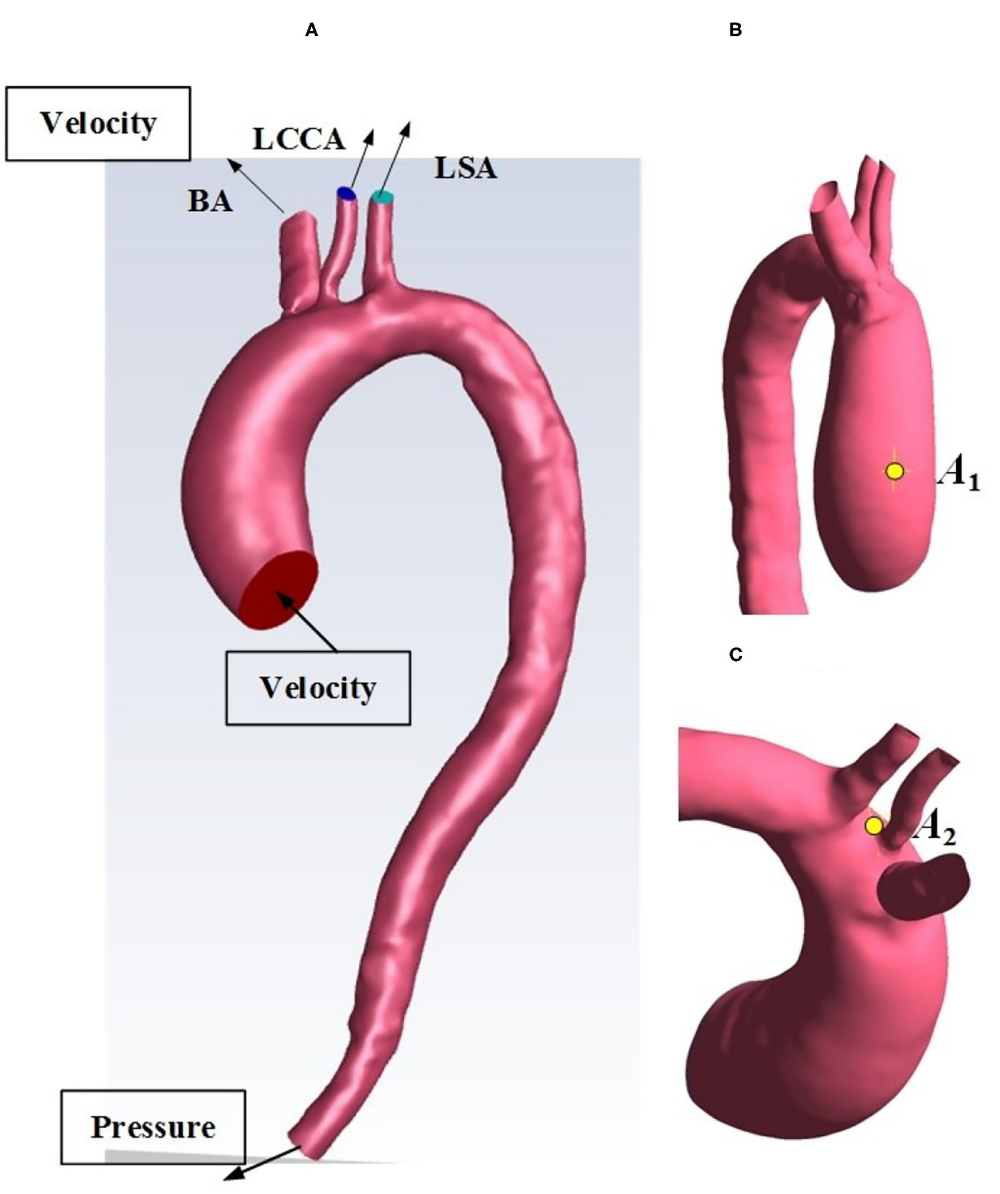
**(A)** Reconstructed geometry of the aortic arch for an HF patient. **(B)** selected location *A*_1_. **(C)** selected location *A*_2_. BA, brachiocephalic trunk; LCCA, left common carotid artery; LSA, left subclavian artery.

The enhanced-CT medical image data used in this study were from The First Affiliated Hospital of Dalian Medical University, China. The subject was an 80-year-old man with valve disease and reduced cardiac output. For the image data, the layer spacing is 0.74 mm, and the plane resolution is 512 × 512. This enhanced CT image data were imported into Mimics 21.0 (Materialise, Leuven, Belgium), a medical image processing software, with DICOM (digital imaging and communications in medicine) format for data processing and three-dimensional (3D) reconstruction, to acquire a geometric model of the patient-specific aorta. Automatic threshold segmentation and manual separation were used to separate the ascending aorta, brachiocephalic trunk, left common carotid artery, left subclavian artery, descending aorta, and abdominal aorta from other tissues. After smooth processing, the 3D reconstruction model of the aortic arch was obtained while maintaining the original physiological and anatomical characteristics, shown in Figure 1A.

### Geometry of Impella CP

**Figure 2:**
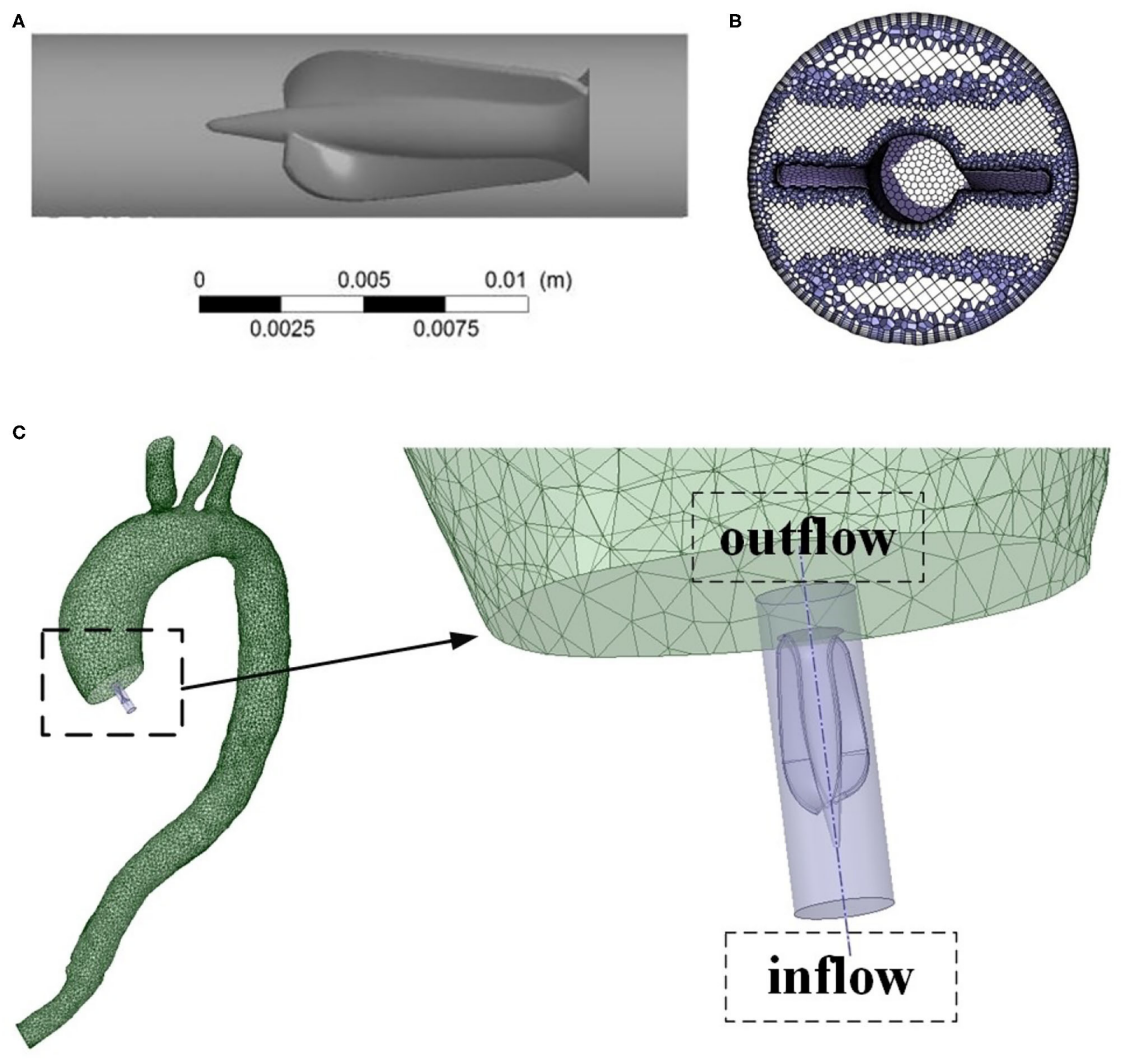
**(A)** Geometric model of the Impella CP. **(B)** Crosssection mesh structure of Impella CP. **(C)** The location of Impella CP and its partially enlarged detail.

The 3D structure model of Impella CP drawn with a 3D software, Proe 2.0 (Parametric Technology Corporation, Boston, MA, USA), crosssection mesh structure of Impella CP, and the location of Impella CP with its partially enlarged detail are shown in Figure 2. The pump body is an impeller, composed of a shaft with a fixed end and double blades with free end, and rotates counterclockwise during operation. Two blades are generated by spiral scanning and parameterization, and the impeller is enclosed in the pump housing. The two bottoms of the cylinder were considered as the inlet and outlet of the housing, respectively. Blood was pumped from the left ventricle into the housing inlet, and flows out of the outlet. Note that the specific geometric sizes and parameters of Impella CP are referred from the literature (Roberts et al., 2020).

### Patient-Specific Aorta Model Without the Impella CP Support

**Figure 3:**
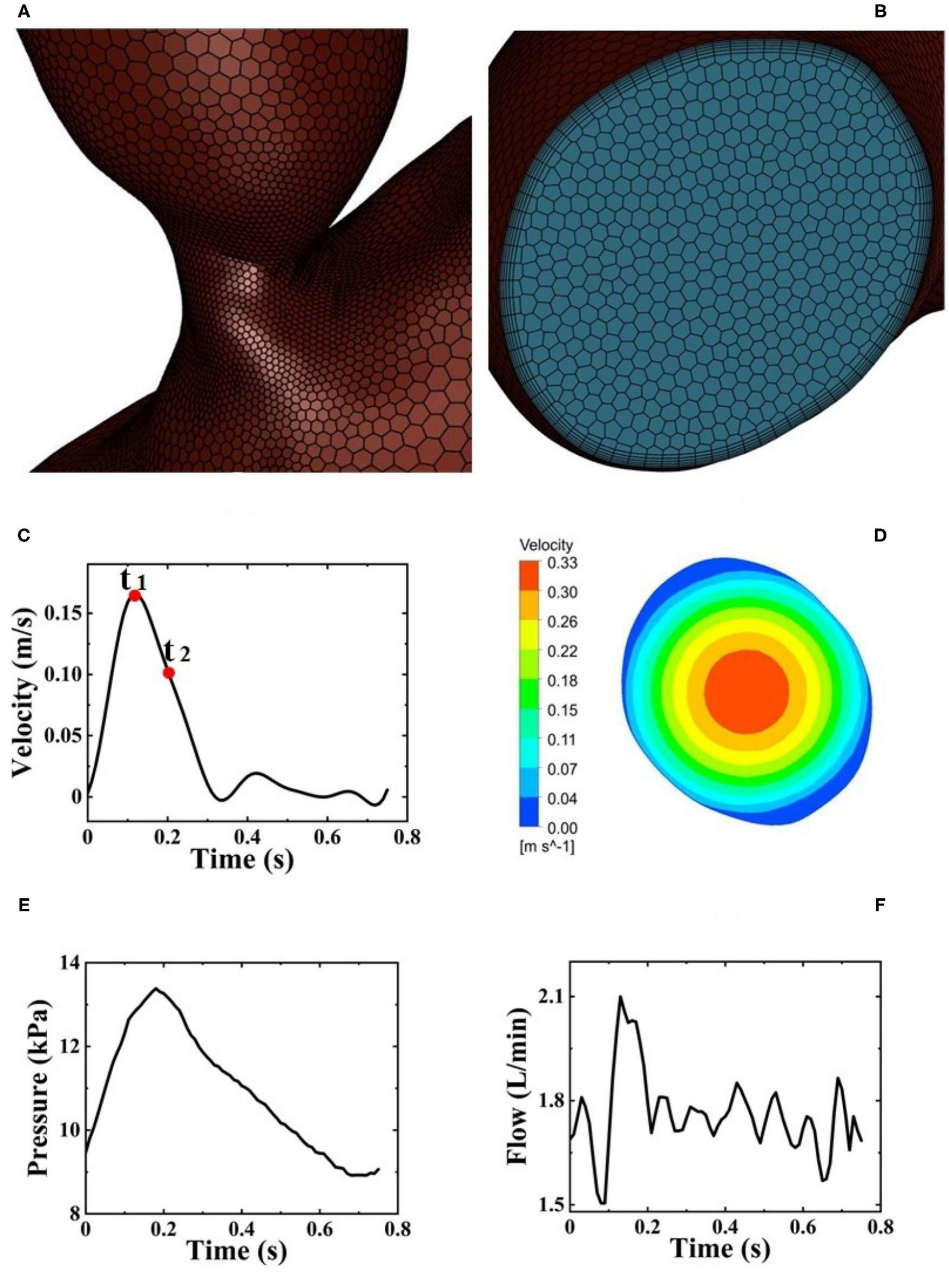
**(A)** Local mesh structure of aortic arch. **(B)** Mesh structure of the inlet of aortic arch. **(C)** Pulsatile blood flow velocity at the inlet of aorta from one HF patient during one cardiac cycle and two selected critical time points *t*_1_ and *t*_2_. **(D)** Velocity distribution of fully developed blood flow perpendicular to the direction of blood flow. **(E)** Pulsatile pressure at the outlet of Impella CP. **(F)** Pulsatile flow rates at the outlet of Impella CP.

The 3D geometric model of the patient-specific aorta was imported into the commercial CFD software package, ANSYS Fluent 2020 R1 (Ansys Inc., Canonsburg, PA, USA) for mesh generation. The domain tetrahedral unstructured meshes of about 850,000 elements were established, partly shown in Figures 3A,B, and the boundary layer was set on the surfaces to meet the constraints of the turbulence model. The fluid part of the grid is imported into ANSYS Fluent 2020 R1 to set boundary conditions. Pulsatile blood velocity at the inlet of aorta is considered as the inlet condition, and the change of blood flow velocity with time is shown in Figure 3C. Note that the velocity waveform used as the inlet condition in this study was obtained from the literature (Estrada et al., 2011). It was scaled in order to make it match the physiological characteristics in the human cardiovascular system such that the baseline flow rate without Impella CP support was less than 3 L/min (Sayago et al., 2015). It is assumed that at the inlet of aorta, the distribution of blood flow velocity, *V*_*in*_, on the crosssection of blood vessels perpendicular to the direction of blood flow is parabolic, whose contour is shown in Figure 3D, and the blood flow velocity in the polar coordinate system is expressed as:


(6) $$\begin{array}{l} V_{in}\left(r,t\right)=2V_{in\text{\_}mean}\left(t\right)[1-{(\frac{2r}{d})}^{2}] \end{array}$$

Note that since the inlet surface is approximately a circular surface, the centroid and area of the inlet surface can be obtained using ANSYS Fluent 2020 R1, and the centroid is regarded as the center of an approximate circular surface. Therefore, in Equation (6), *r* represents the distance from the approximate center and *d* represents the approximate diameter calculated based on the area of inlet surface, **V**_*in*_*mean*_ is the average velocity on the crosssection. The above mathematical expressions for calculating the required waveform of blood flow velocity was written in C-language with user-defined functions (UDFs), which can be compiled and loaded into the fluent library. Zero pressure at the outlet of aorta is prescribed as the outlet condition (Zhang J. et al., 2020).

In addition, at present, it is difficult to obtain blood flow data in the three branches of aorta such as brachiocephalic trunk, left common carotid artery, and left subclavian artery on the basis of the individual patients with various cardiovascular diseases. Therefore, in this study, the sum of blood flow from the above three branches accounted for 30% (Chi et al., 2017) out of the total flow rates. In the meanwhile, the ratio of flow rates from brachiocephalic trunk, left common carotid artery, and left subclavian artery was set as 1.5:1:1.2 (Chi et al., 2017), respectively. As a result, the blood flow velocity of brachiocephalic trunk, left common carotid artery, and left subclavian artery can be calculated based on the crosssection areas of the above three branch outlets. In addition, while the three branches are set as velocity inlets, they have a negative inlet velocity to represent the outflows out of the three arch vessels (Hu et al., 2020). Furthermore, in this study, we assumed that there were no additional branching vessels in the aorta downstream of the three main aortic branches to simplify the CFD simulation.

#### Patient-Specific Aorta Model During the Impella CP Support

First, after using Proe 2.0 to establish the 3D geometric model of Impella CP, ANSYS Fluent 2020 R1 is used to mesh the flow field of Impella CP, resulting in the domain tetrahedral unstructured meshes of about 290,000 elements. Pulsatile velocity and pressure are set as the boundary conditions at the inlet and outlet of Impella CP (Estrada et al., 2011; Roberts et al., 2020), respectively, as shown in Figures 3C,E. The impeller rotates at a constant speed of 46,000 r/min with a sliding mesh. Second, during one cardiac cycle, the total flow rate is calculated as the sum of the flow rate of the outlet of Impella CP (Figure 3F) and the native flow rate generated by the heart. The total flow rate is used to calculate the pulsatile velocity in the aorta (inlet boundary condition) using the inlet area of the aortic arch. The other settings are the same as those for the patient-specific aorta model without Impella CP.

### Numerical Schemes and Procedures

The semiimplicit method for pressure linked equations pressure–velocity method was used to solve the above fluid governing equations. The spatial discretization schemes of gradient, pressure, and momentum are based on least square cell, standard, and second-order upwind, respectively. Aorta surfaces and walls of Impella CP are set to the no-slip condition. Moreover, in this study, two representative time points *t*_1_ and *t*_2_ (Figure 3C) (Chi et al., 2017) and two critical locations *A*_1_ and *A*_2_ (Figures 1B,C) are selected for hemodynamic analysis. In detail, *t*_1_ is chosen at 0.12 s as the peak systole, while *t*_2_ is chosen at 0.22 s as the representative point during the deceleration ejection period. The locations *A*_1_ and *A*_2_ are near the ascending aorta and bifurcating area, respectively, because clinically the outflow of Impella CP is at the ascending aorta (near *A*_1_) and the sharp geometric changes could easily happen at the bifurcating areas (near *A*_2_). Some WSS and pressure waveforms were obtained using the “chart” function in CFD-post of ANSYS Fluent 2020 R1.

### Mesh and Time Independence Test

**Figure 4:**
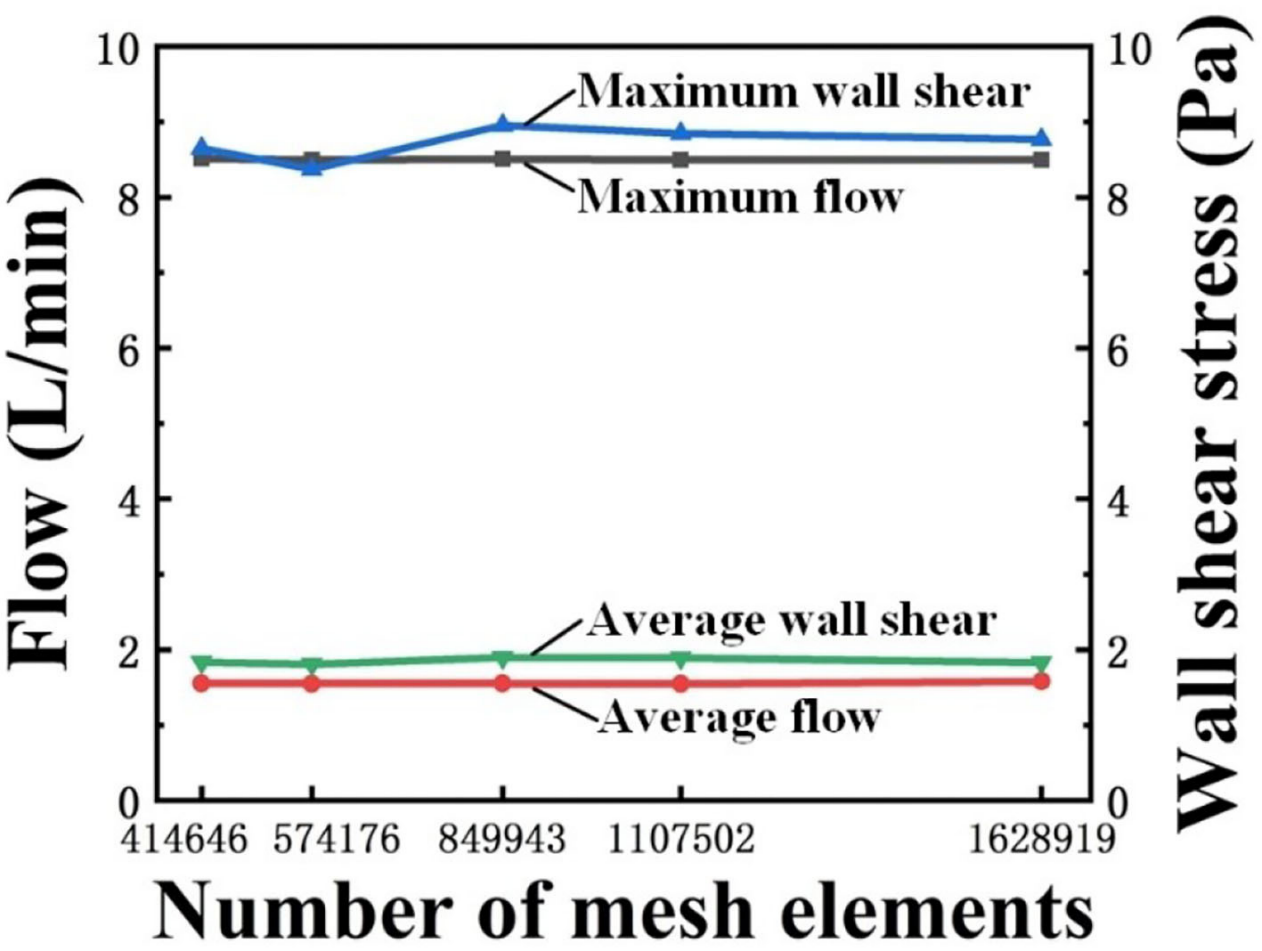
Mesh independent verification results.

Mesh independence test in this study was implemented by using five different meshes with 414,646; 574,176; 849,943; 1,107,502; and 1,628,919 elements, respectively. The convergence criterion was set as 0.0001. The mesh had a negligible effect on the calculation results when the number of elements in the mesh was >849,943, as shown in Figure 4. Therefore, to optimize between computational cost and accuracy, 849,943-element mesh was selected.

The time steps were initially selected as 0.01 and 0.005 s, respectively, for the time independence study. Under the two time-step conditions, the difference between the outlet velocity and WSS distribution was small (<1%); however, the computational cost significantly increased when the time step of 0.005 s was used. As a result, a time step of 0.01 s was used in this study and the total calculation time was 30.5 h. In addition, the simulations were conducted for six cardiac cycles to eliminate the initialization effect and achieve stable results. The data during the last cycle was used for analysis (Xiong et al., 2020).

## Results

### Flow Patterns

**Figure 5:**
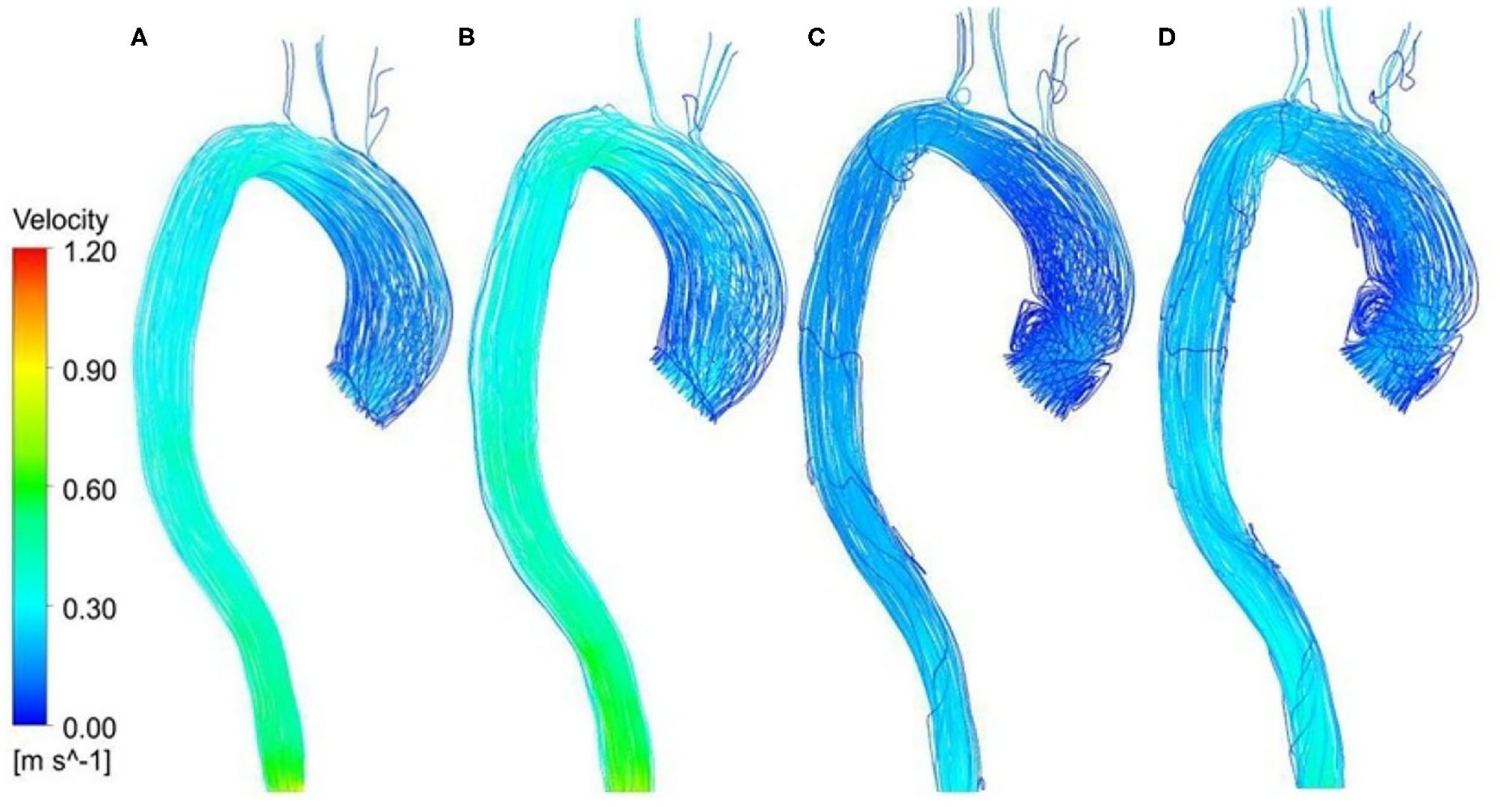
Velocity streamlines at *t*_1_ for the patient-specific aorta model **(A)** without Impella CP support, **(B)** with Impella CP support, and velocity streamlines at *t*_2_ for the patient-specific aorta model **(C)** without Impella CP support, and **(D)** with Impella CP support.

**Figure 6:**
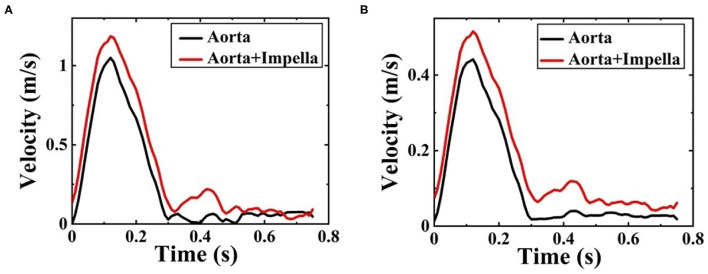
**(A)** The center velocity at aortic arch outlet during one cardiac cycle without and with the Impella CP support and **(B)** the average velocity in the aortic arch during one cardiac cycle without and with the Impella CP support.

Figures 5A,B shows a comparison of velocity streamlines during peak systole at *t*_1_ for the patient-specific aorta model without and with the Impella CP assistance, respectively, and the velocity streamlines during the slow ejection period at *t*_2_ for the patient-specific aorta model without and with the Impella CP assistance is shown in Figures 5C,D, respectively. Apparently, the flows in this HF patient-specific aorta model were fairly organized. Obvious swirls were observed in the upstream of the aortic valve outlet during the slow ejection period corresponding to *t*_2_ in Figures 5C,D. Figure 6A presents the center velocity at the aortic arch outlet during one cardiac cycle without and with the pump, respectively. The peak velocity is 1.05 m/s without the pump compared with that of 1.19 m/s with the pump. Figure 6B displays the average velocity in the aortic arch during one cardiac cycle without and with the Impella CP, respectively. The peak velocity is 0.44 m/s without the Impella CP support and it increased to around 0.52 m/s with the Impella CP support. In addition, the average aortic flow without the pump is 2.6 L/min, which is increased to 3.9 L/min with the pump assistance.

### Wall Shear Stress

**Figure 7:**
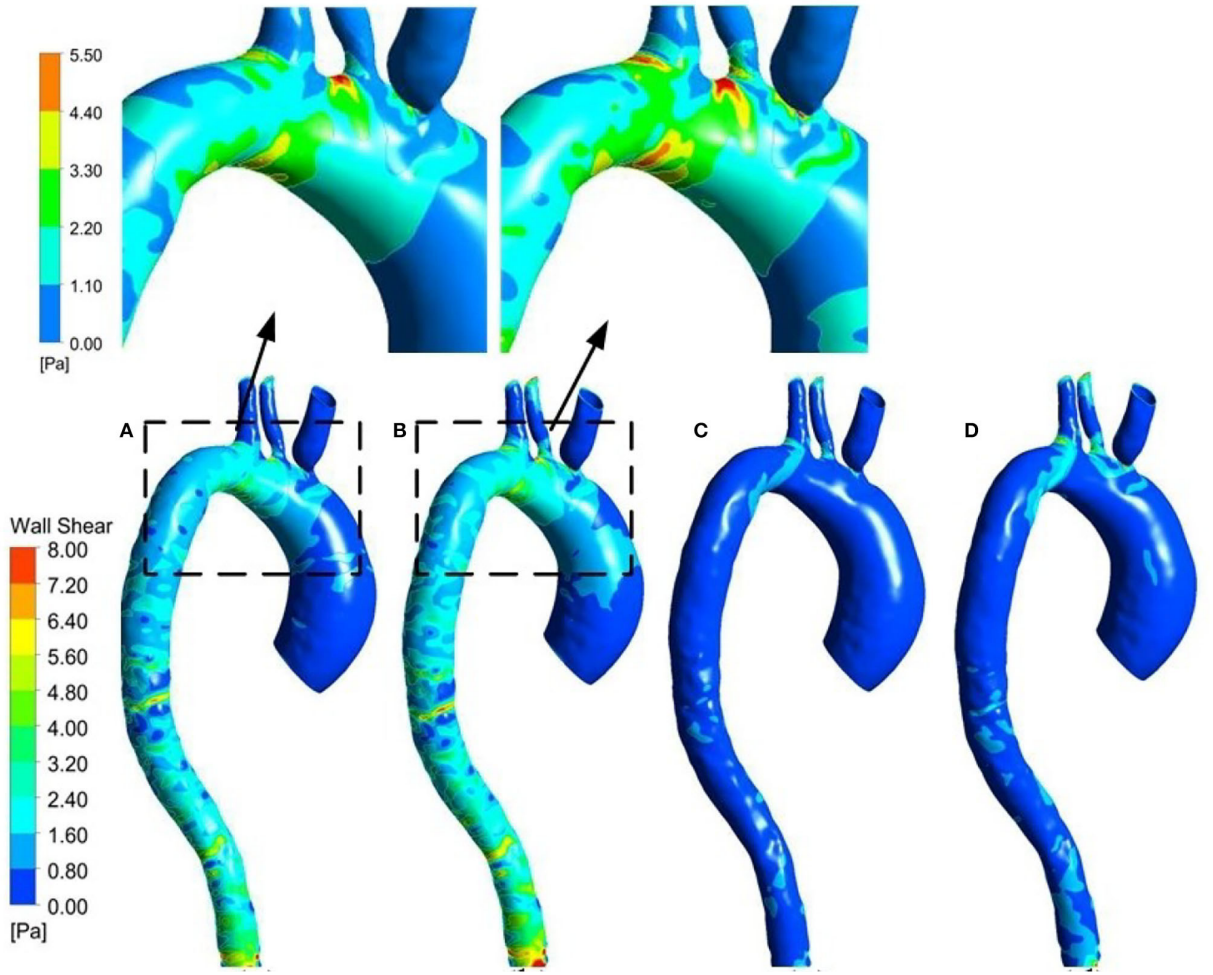
WSS distribution at *t*_1_ for the patient-specific aorta model **(A)** without Impella CP support, **(B)** with Impella CP support, and WSS distribution at *t*_2_ for the patient-specific aorta model **(C)** without Impella CP support, and **(D)** with Impella CP support. The two top subfigures are rescaled and enlarged based on the dashed parts in **(A,B)**, respectively.

**Figure 8:**
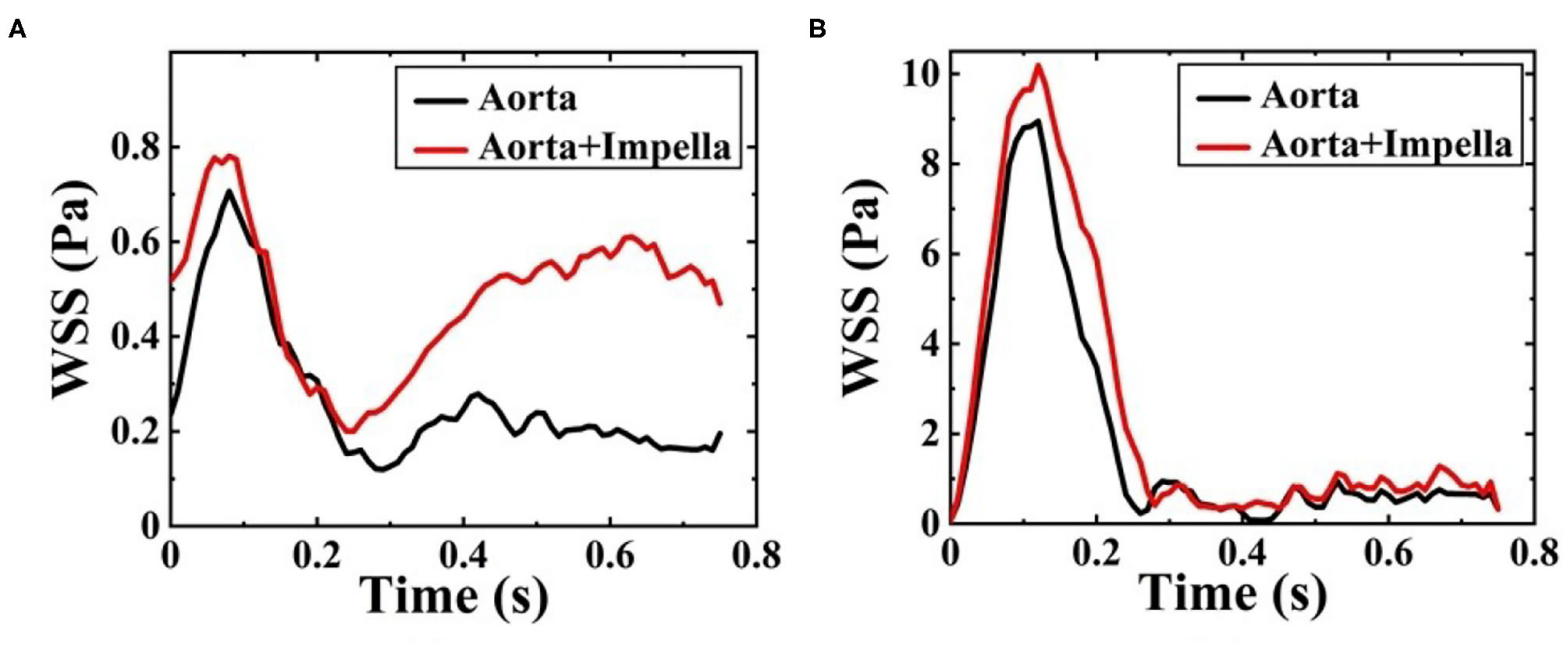
Waveform of WSS at **(A)** *A*_1_ and **(B)** *A*_2_ during one cardiac cycle without and with Impella CP.

The WSS distribution on the inner surface of the patient-specific aorta model at time *t*_1_ and *t*_2_ for one cardiac cycle without and with the Impella CP support is shown in Figure 7. Once the effects of Impella CP are considered, WSS values at most locations especially near the descending aorta and bifurcating areas would be larger than those without Impella CP support, regardless of the two selected representative time points. In addition, it is usually difficult to use an *in vivo* way to directly measure WSS, which is an extremely important hemodynamic quantity. Thus, in order to obtain the information of high WSS on the patient-specific aorta model, analyzing the WSS distribution patterns was conducted ahead of time. To more clearly observe the increased WSS in other regions, in this work, the maximum WSS value has been rescaled as decreased to 5.5 Pa and the upside of the aortic arch is enlarged, which is shown in Figure 7. Figure 8 shows the waveform of WSS at two selected locations *A*_1_ and *A*_2_. Clearly, the value of WSS without Impella CP support was lower than that with Impella CP support regardless of the two locations, and the maximum value of WSS at *A*_2_ is much higher than that at *A*_1_ regardless of the Impella CP support.

### Δ*P* Distribution

**Figure 9:**
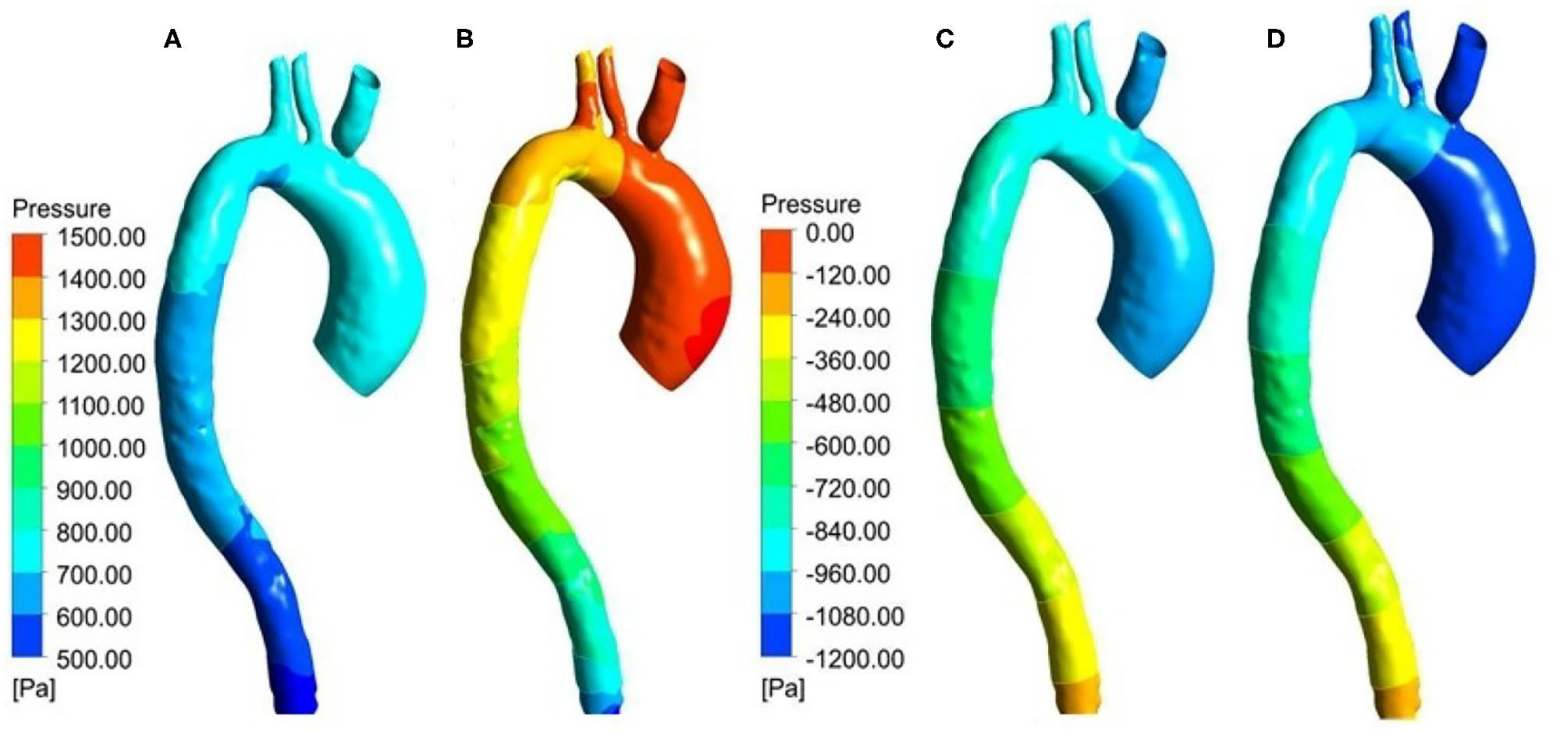
Pressure distribution at *t*_1_ for the patient-specific aorta model **(A)** without Impella CP support, **(B)** with Impella CP support, and pressure distribution at *t*_2_ for the patient-specific aorta model **(C)** without Impella CP support, and **(D)** with Impella CP support.

**Figure 10:**
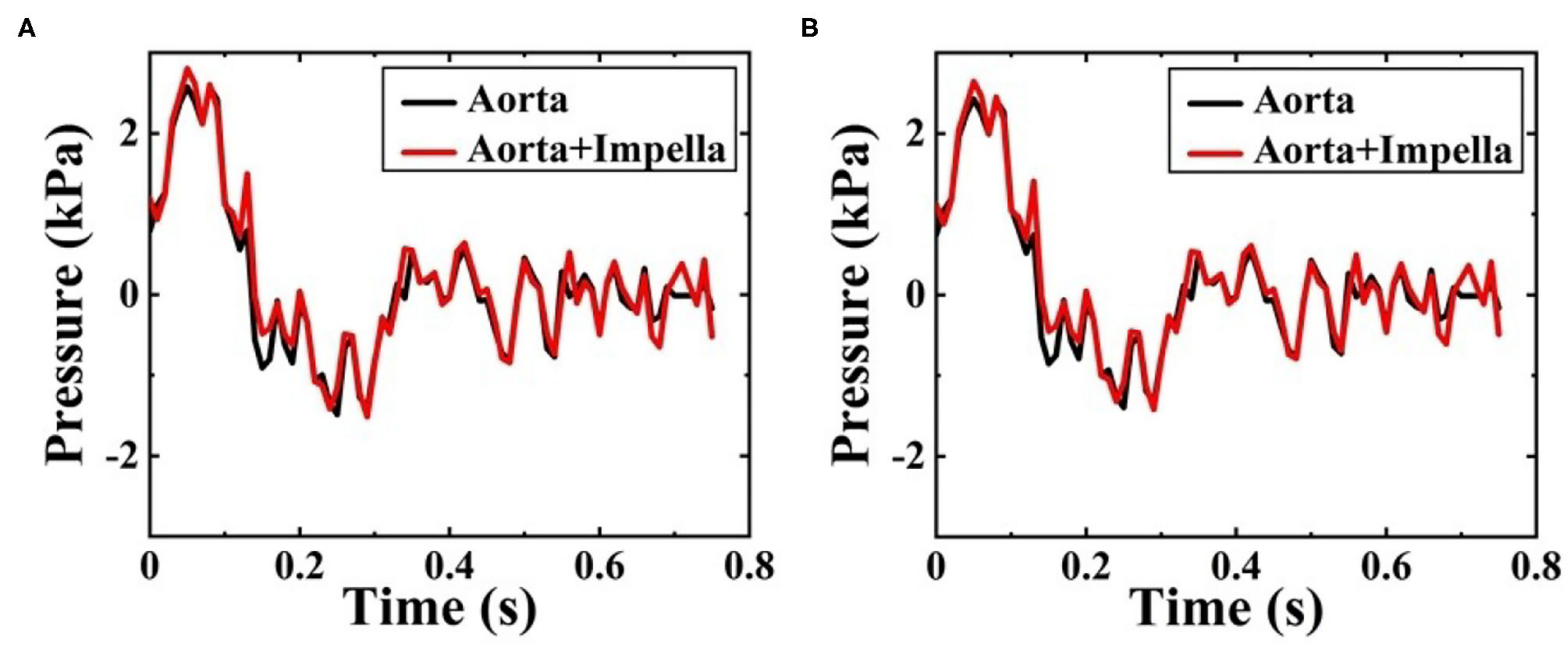
Pressure waveforms at **(A)** *A*_1_, and **(B)** *A*_2_ during one cardiac cycle without and with the Impella CP assistance.

In this study, Δ*P* means the difference between the pressure at any point on the vessel walls and the outlet pressure. Figures 9A,B shows the Δ*P* contour map of patient-specific aorta model at time *t*_1_ during systolic period without Impella CP and during Impella CP support, respectively. Δ*P*-values of the vessel wall are higher during Impella CP support than those without Impella CP support, and Δ*P*-values decrease gradually from the ascending aorta to the distal aorta. However, the opposite phenomenon was found at time *t*_2_ during the deceleration ejection period without and with the Impella CP support, respectively, which is shown in Figures 9C,D such that Δ*P*-values of vessel wall gradually increased from the ascending aorta to the distal aorta. Furthermore, Figure 10 shows Δ*P* waveforms at two selected locations *A*_1_ and *A*_2_. For *A*_1_, the maximum Δ*P*-value was 2.6 kPa without Impella CP support and increased to 2.8 kPa, whereas the effects of Impella CP were considered. For *A*_2_, the maximum Δ*P*-values were 2.4 and 2.6 kPa without and with the Impella CP support, respectively. Note that in Figure 10, the two waveforms are extremely similar. The reason is that the distance between the selected two representative points *A*_1_ and *A*_2_ are very close, causing the pressure waveforms at *A*_1_ and *A*_2_ to be very similar.

## Discussion

Computational fluid dynamics simulation could effectively display the local hemodynamic effects in the cardiovascular system and enable assessments of the effects of LVADs in patients. In this numerical study, the proposed CFD modeling focused on the hemodynamic effects of a short-term Impella CP on a patient-specific aorta model, and was compared with the baseline results without Impella CP support. The numerical results demonstrated the changes inside the aorta, including flow velocity, WSS, and Δ*P*. These data may help optimize the device and treatment of HF patients using short-term LVADs.

The disturbed flow that occurs near the inlet of aorta at *t*_2_ in Figures 5C,D may be due to the slow velocity at this moment (Mazzitelli et al., 2016). Comparatively, such disturbed flow was not found in Figures 5A,B in the ascending aorta at *t*_1_. The peak value of averaged velocity in the aortic arch in Figure 6B is lower than that of center velocity at aortic arch outlet in Figure 6A because the velocity is inversely proportional to the area. The center velocity at aortic arch outlet, the average velocity in the aortic arch, and the average aortic flow rates with the Impella CP support are higher than those without the Impella CP support.

In this CFD modeling, the boundary conditions used pulsatile velocity and pressure at the inlet and outlet of Impella CP, respectively. However, the rotational speed of Impella CP was held constant. It has been proven that LVAD support diminished (nonphysiological) arterial pulsatility. Diminished pulsatility has been associated with adverse events in peripheral blood vessels and other organs, like arteriovenous malformation, hemorrhagic stroke, and damage to other organs such as kidney (Soucy et al., 2013; Ross et al., 2018; Cho et al., 2019). Even short-term diminishment of pulsatility has been associated with endothelial dysfunction (Nguyen et al., 2021). In contrast, LVADs with pulsatile pump speed mode could improve arterial pulsatility and did not further lead to the risk of blood damage (Chen et al., 2018; Haglund et al., 2019). Therefore, one of the future works is to study the quantitative relationship between the modulated pump speed operation of short-term LVADs anastomosed to the patient-specific aorta model and key hemodynamic signals.

As one of the most important hemodynamic parameters, WSS is related to the vascular and arterial adaptation. The simulation results showed that changes in WSS with and without the Impella CP support at two selected representative locations *A*_1_ and *A*_2_ were obvious. WSS values on the aorta were much lower than those within the Impella CP. The highest shear stresses were generated at the impeller tips when the Impella CP is rotating at a very high speed (~46,000 rpm). Comparatively, WSS in the flow field is related to the blood velocity, which cannot be as high as that at the impeller tip. In general, reduction of WSS within the LVADs is beneficial as it reduces the potential risk of blood trauma, which remains an important clinical concern. In addition, with reference to the Δ*P* analysis, it is possible to note that Δ*P* was usually larger than WSS. Especially at *t*_1_, the peak systole, blood increased and flowed *via* the ascending aorta with high Δ*P*, which expanded the arterial wall due to the compliance of the aorta.

This study was still subject to some limitations. For example, these results were specific to one clinical patient-specific aorta data, which ensured that the individual effects of the LVAD support can be analyzed on a patient specific basis. However, global predictions of the LVAD support on aorta required averaging of aortic dimensions on a larger cohort of patients. The aortic model did not consider small aortic branch vessels distal to the aortic arch vessels that can cause additional outflows, and this CFD study did not analyze measures of hemolysis including hemolysis index. Moreover, in this research we only studied the changes in the local hemodynamic effects of aortic arch before and after implanting Impella CP, and zero pressure was used as the outlet condition, implying that the characteristics of downstream vascular system of aortic arch were not considered in the CFD simulation by using the Windkesel model with boundary conditions of proximal resistance and capacitance, and distal resistance (Xiong et al., 2020), which may cause negative pressure and dramatic changes of Δ*P* in Figure 10. One of the future work is to reset the boundary conditions of outlet by considering the factors of downstream vascular system of aortic arch to make the CFD simulation results more consistent with the clinical results. Despite these limitations, the proposed study demonstrated the local hemodynamic changes in the aorta with short-term LVAD support devices.

## Conclusion

In this paper, a quantitative CFD simulation was conducted for studying the local hemodynamic effects of a short-term LVAD in a patient-specific aorta model. Velocity, WSS, and Δ*P* in the aorta were calculated without and during the Impella CP support for comparison. Results demonstrated that Impella CP support augmented velocity, WSS, and Δ*P* within the aorta. Future study is needed using more effective and accurate CFD modeling, and with larger number of subjects and pulsatile pump speed conditions.

## Data Availability Statement

The original contributions presented in the study are included in the article/supplementary material, further inquiries can be directed to the corresponding author/s.

## Ethics Statement

Ethical review and approval was not required for the study on human participants in accordance with the local legislation and institutional requirements. Written informed consent for participation was not required for this study in accordance with the national legislation and the institutional requirements.

## Author Contributions

YW: methodology, writing—original draft preparation, and editing. JW: software and writing—original draft preparation. JP and MH: software. ZY: data acquisition, validation, and formal analysis. GG: writing—reviewing and supervision. YL and KQ: writing—reviewing, editing, and supervision. All authors contributed to the article and approved the submitted version.

## Funding

This work was supported, in part, by the National Natural Science Foundation of China (Grant Nos. 32071314 and 31971243), LiaoNing Revitalization Talents Program of China (Grant No. XLYC1807016), and National Institutes of Health R01 grant (Grant No. 1R01HL150346).

## Conflict of Interest

The authors declare that the research was conducted in the absence of any commercial or financial relationships that could be construed as a potential conflict of interest.

## Publisher's Note

All claims expressed in this article are solely those of the authors and do not necessarily represent those of their affiliated organizations, or those of the publisher, the editors and the reviewers. Any product that may be evaluated in this article, or claim that may be made by its manufacturer, is not guaranteed or endorsed by the publisher.
